# Pomegranate-Like Structured Si@SiO_x_ Composites With High-Capacity for Lithium-Ion Batteries

**DOI:** 10.3389/fchem.2020.00666

**Published:** 2020-09-11

**Authors:** Jianbin Li, Wenjing Liu, Yingjun Qiao, Gongchang Peng, Yurong Ren, Zhengwei Xie, Meizhen Qu

**Affiliations:** ^1^Department of Nano Carbon Materials, Chengdu Institute of Organic Chemistry, Chinese Academy of Sciences, Chengdu, China; ^2^Group of Chemistry and Chemical Engineering, University of Chinese Academy of Sciences, Beijing, China; ^3^Jiangsu Collaborative Innovation Center of Photovoltaic Science and Engineering, School of Materials Science and Engineering, Changzhou University, Changzhou, China

**Keywords:** lithium-ion battery, anode, silicon, pomegranate-like structured, spray drying

## Abstract

Silicon anodes with an extremely high theoretical specific capacity of 4,200 mAh g^−1^ have been considered as one of the most promising anode materials for next-generation lithium-ion batteries. However, the large volume expansion during lithiation hinders its practical application. In this work, pomegranate-like Si@SiO_x_ composites were prepared using a simple spray drying process, during which silicon nanoparticles reacted with oxygen and generated SiO_x_ on the surface. The thickness of the SiO_x_ layer was tuned by adjusting the drying temperature. In the unique architecture, the SiO_x_ which serves as the protection layer and the void space in pomegranate-like structure could alleviate the volume expansion during repeated lithium insertion/extraction. As a lithium-ion battery anode, pomegranate-like Si@SiO_x_ composites dried at 180°C delivered a high specific capacity of 1746.5 mAh g^−1^ after 300 cycles at 500 mA g^−1^.

## Introduction

Lithium-ion batteries (LIBs) have been widely used in portable electronics, electric vehicles, and large-scale energy storage systems owing to their high energy density, long cycling stability, and environmental friendliness (Armand and Tarascon, 2008; Yu et al., 2018; Zuo et al., 2019; Kwon et al., 2020; Li et al., 2020; Wang K. et al., 2020). Nowadays, the traditional commercial anode graphite (372 mAh g^−1^) is not able to meet increasing demands for higher energy density. In this context, a Si anode with an ultra-high theory specific capacity (4,200 mAh g^−1^) and lower work potential (<0.5 V vs. Li^+^/Li) have emerged as an alternative (Szczech and Jin, 2011; Zhuang et al., 2017; Zuo et al., 2017; Fang et al., 2020a). However, during the lithiation process, the Si anode undergoes large volume expansion (~320%, Li_22_Si_5_), which leads to active material pulverizing and splitting away from the current. A solid electrolyte interface (SEI) for a Si anode is unstable, resulting in fracture and regeneration during the charge/discharge process. The SEI layer continuously grows and irreversibly consumes lithium-ions in the electrolyte, leading to inferior lithium-ion kinetics and poor electrochemical performance. Moreover, the Si anode is a semiconductor with poor conductivity, which is not conducive to delivering capacity (Zheng et al., 2019; Zuo et al., 2019; Kwon et al., 2020).

Many efforts have been made to solve these limitations. Studies have shown that nanostructures including 0D nanoparticles (Xu Y. et al., 2017), 1D nanowires/nanotubes (Ge et al., 2012; Kennedy et al., 2017), 2D nanofilms, and 3D porous structures (Yang et al., 2015; Xu et al., 2017; Chen et al., 2018; Mu et al., 2019; Fang et al., 2020b) can significantly reduce the volume effect and shorten the diffusion distance of lithium-ion. Combining Si with other matrices is also an efficient alternative way to alleviate the volume expansion stress (Cheng et al., 2018; Jo et al., 2019; Tao et al., 2019). Mu et al. used self-assembly and magnesiothermic reduction to prepare a pomegranate-like core-shell structured Si@C composite (Mu et al., 2019). Benefiting from the void space in the special architecture, the Si@C anode delivered a reversible capacity of 1,494 mAh g^−1^ after 100 cycles at 100 mA g^−1^, corresponding to an outstanding capacity retention of 97% (He et al., 2017). In another work, Kim et al. creatively obtained Si/SiO_x_ from a sol-gel reaction using trithoxysilane as the precursor. After coating it with a uniform carbon film, it maintained a discharge capacity of 740 mAh g^−1^ after 100 cycles at 200 mA g^−1^. This satisfactory performance is mainly due to the amorphous SiO_x_ matrix, which can alleviate the volume expansion during the charge/discharge process (Park et al., 2014). During the lithiation process, SiO_x_ reacts with Li^+^ to generate Li_2_O and a series of lithium silicates, which favors the construction of a stable SEI (Song et al., 2013; Wang et al., 2014; Liu et al., 2019).

(1) $$\begin{array}{r} SiO_{x}+2xLi^{+}+2xe^{-}\to Si+xLi_{2}O \end{array}$$

(2) $$\begin{array}{r} SiO_{x}+yLi^{+}+ye^{-}\to Li_{y}SiO_{x} \end{array}$$

Based on the above discussion, by structural design and introducing SiO_x_ (silicon oxide, 0 < x < 2) as the protection layer, the volume expansion stress of a Si anode can be effectively alleviated. In this study, we developed pomegranate-like Si@SiO_x_ second particles by a one-step spray drying process, during which silicon nanoparticles reacted with oxygen and produced a uniform SiO_x_ protection layer. The unique pomegranate-like structure also contained a number of voids that could further maintain structural stability during cycling. We believe that this unique pomegranate-like Si@SiO_x_ could provide excellent electrochemical performance in LIBs.

## Experimental Section

### Synthesis of Pomegranate-Like Structured Si@SiO_x_ Composites

Pomegranate-like structured Si@SiO_x_ composites were fabricated through simple spray drying using nano-silicon as the precursor. Typically, 3 g of silicon nanoparticles (SiNPs, 20–120 nm, Guangzhou Hongwu Material Technology Co., Ltd) was added to 300 mL of deionized water and sonicated for 30 min. The homogeneous suspension was then pumped into the spray dryer at a speed of 10 mL min^−1^ and dehydrated using hot air. During the drying process, the fresh surface of the SiNPs reacted with oxygen in the hot air. By adjusting the drying temperature to 150, 180, and 210°C, the thickness of the SiO_x_ layer was tuned, and the Si@SiO_x_ composites were denoted as pSi-150, pSi-180, and pSi-210, respectively.

### Materials Characterization

The morphology and structure of the prepared Si@SiO_x_ were observed using scanning electron microscopy (SEM, JEOL JSM-7500F) and transmission of electron microscopy (TEM, Tecnai G2 F20 S-TWIN). The crystal structure of Si@SiO_x_ was characterized by X-ray diffraction (XRD) using X'pert MPD DY1219 diffractometer (copper Kα source, 40 kV, 20 mA), and the 2θ angle was from 15 to 85° with scanning steps of 0.02°/s. The elemental distribution was analyzed by energy dispersive X-ray spectroscopy (EDS) attached to SEM. X-ray photoelectron spectroscopy (XPS) was performed on a PHI 5,600 physical electronic device. Raman spectra were recorded on LabRAM HR with a laser wavelength of 532 nm, and Fourier transform infrared spectroscopy (FT-IR) was conducted on KBr pellets. The specific surface area and pore size distribution of the sample were measured using nitrogen adsorption-desorption measurements on a Kubo-X1000 spectrometer.

### Electrochemical Measurement

The CR2032 coin cell was used to test the electrochemical performance of the Si@SiO_x_ composites. First, active material pSi composite, conductive agent SP, and binders including sodium carboxymethyl cellulose (CMC), and styrene-butadiene rubber (SBR) were mixed at a mass ratio of 80: 10: 4: 6, respectively. Then, the mixture was ground for 1 h in an agate mortar and coated on copper foil (Wason Copper Foil Co., Ltd). After drying in a vacuum oven at 60°C for 1 h, the copper with anode material was cut into pieces with a diameter of 12 mm and finally dried at 105°C for 12 h in a vacuum oven. Lithium metal was used as the counter electrode. 1 M LiPF_6_ was dissolved in the solution of ethylene carbonate (EC), dimethyl carbonate (DMC), and ethyl methyl carbonate (EMC) in a volume ratio of 1:1:1 as electrolyte. In addition, 2% fluoroethylene carbonate (FEC) was added as the electrolyte additive. Eventually, the coin cell was assembled in an Ar-filled glove box (Chengdu Derek Co., Ltd). The cycling performance and rate capacity of the newly prepared cells were tested on the Neware 4000s in a potential window from 0.01 to 2 V vs. Li^+^/Li. Cyclic voltammetry (CV) and electrochemical impedance spectroscopy (EIS) tests were performed on an electrochemical workstation (Multi Autolab M204, Metrohm). The CV scan region was 0.001–3 V with a sweep speed of 0.1 mV s^−1^ and the EIS measurement ranged from 10 mHz to 100 kHz with an amplitude of 5 mV.

## Results and Discussion

**Figure 1:**
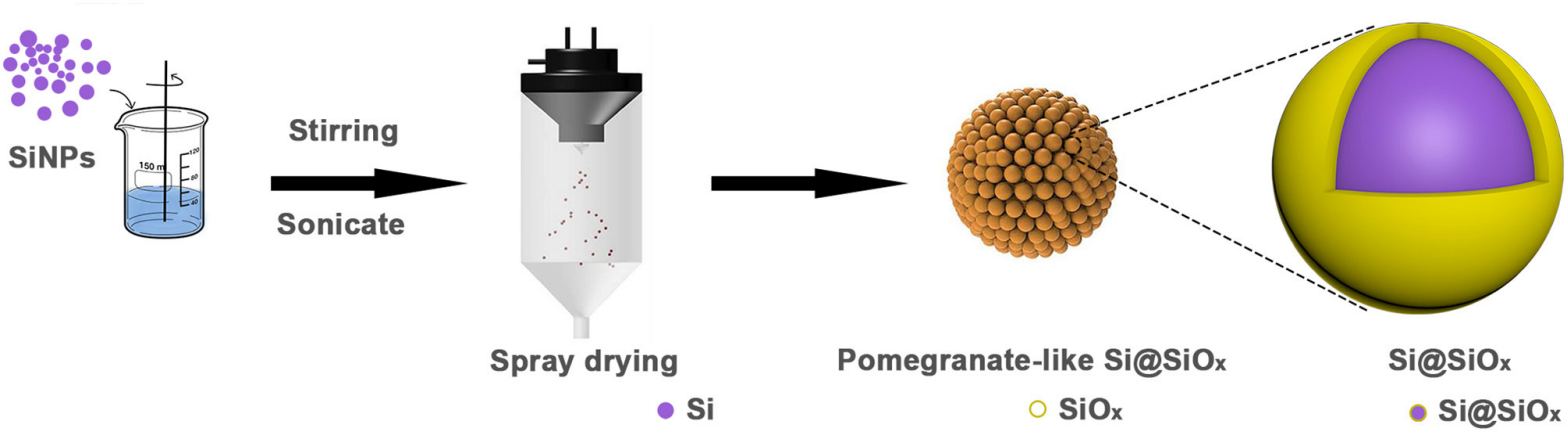
The preparation process of pomegranate-like Si@SiO_x_ composite.

The preparation process of the pomegranate-like structured Si@SiO_x_ composite is illustrated in Figure 1. First, Si nanoparticles were dispersed in an aqueous solution with stirring and sonication. Subsequently, the homogeneous slurry was spray dried at various temperatures, during which the surface of the SiNPs was partly oxidized to SiO_x_ and rearranged to the pomegranate-like structured material.

**Figure 2:**
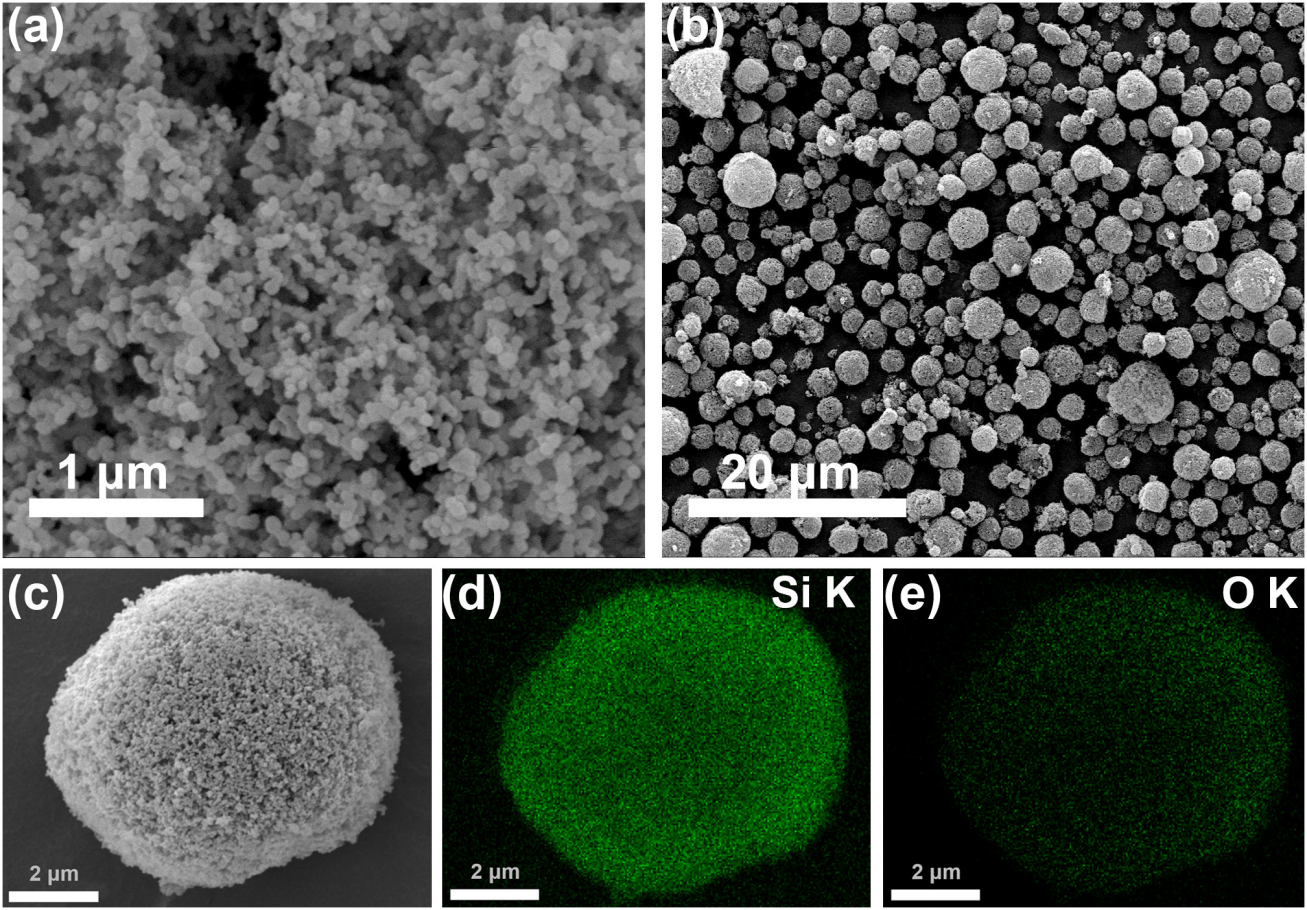
SEM images of SiNPs **(a)** and pSi-180 **(b,c)**; **(d,e)** EDS elemental mapping images of Si and O in pSi-180.

**Figure 3:**
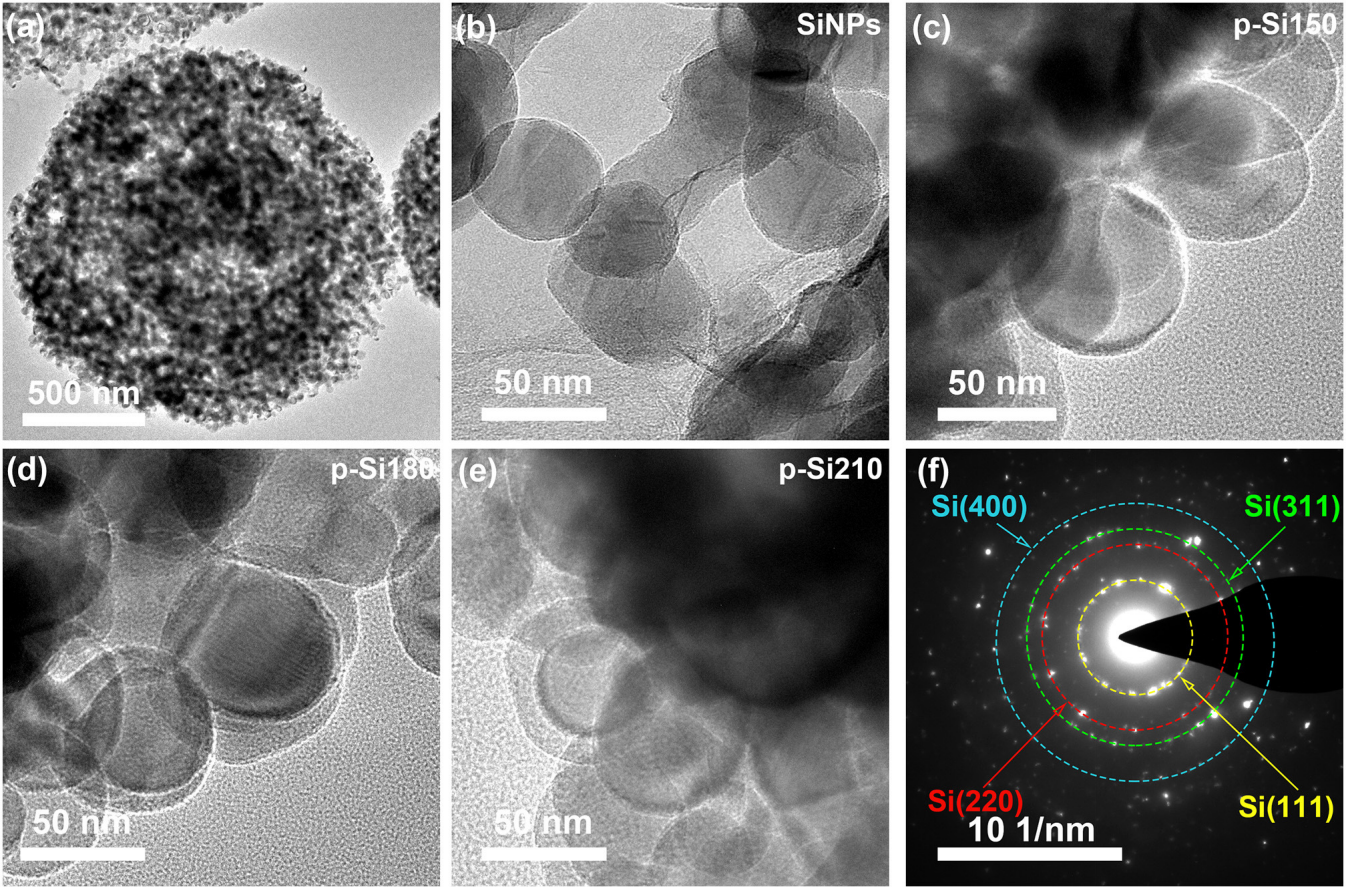
TEM images of SiNPs **(b)**, pSi-150 **(c)**, pSi-180 **(a,d)**, and pSi-210 **(e)**; **(f)** SAED pattern of pSi-180.

**Figure 4:**
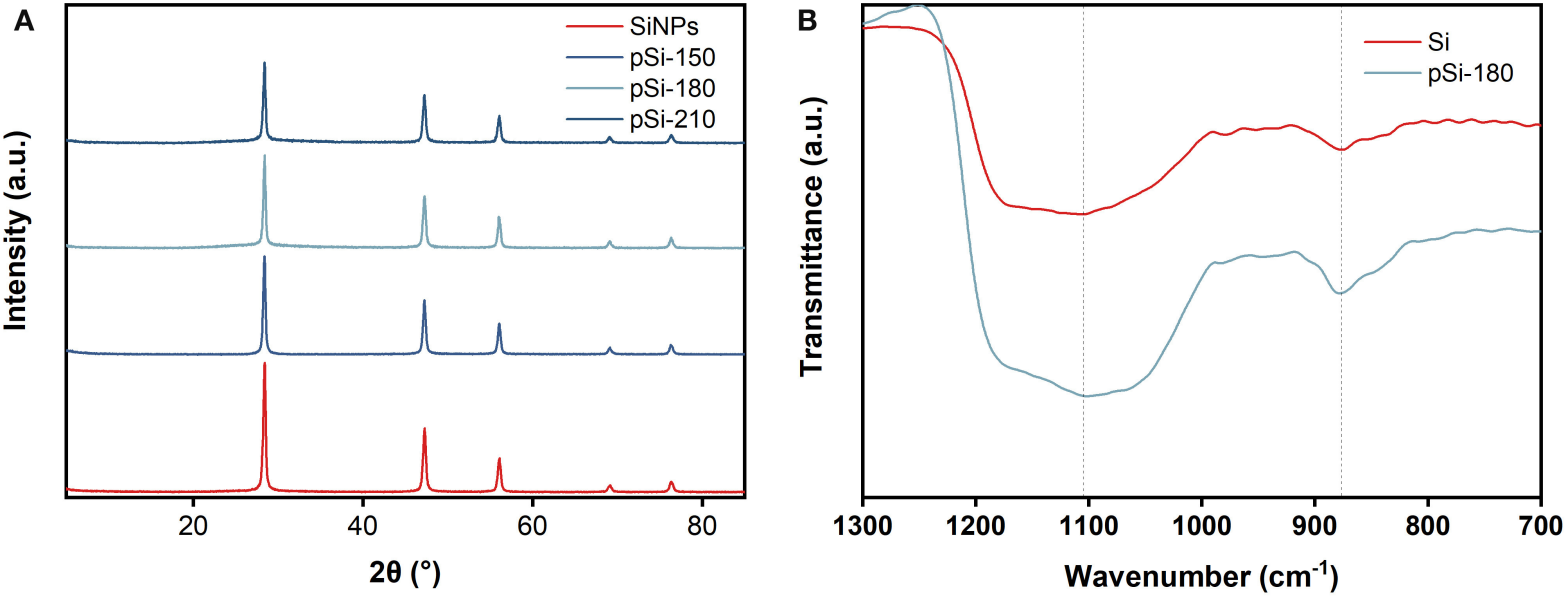
**(A)** XRD patterns of SiNPs, pSi-150, pSi-180, and pSi-210; **(B)** FT-IR spectra of SiNPs, and pSi-180.

**Figure 5:**
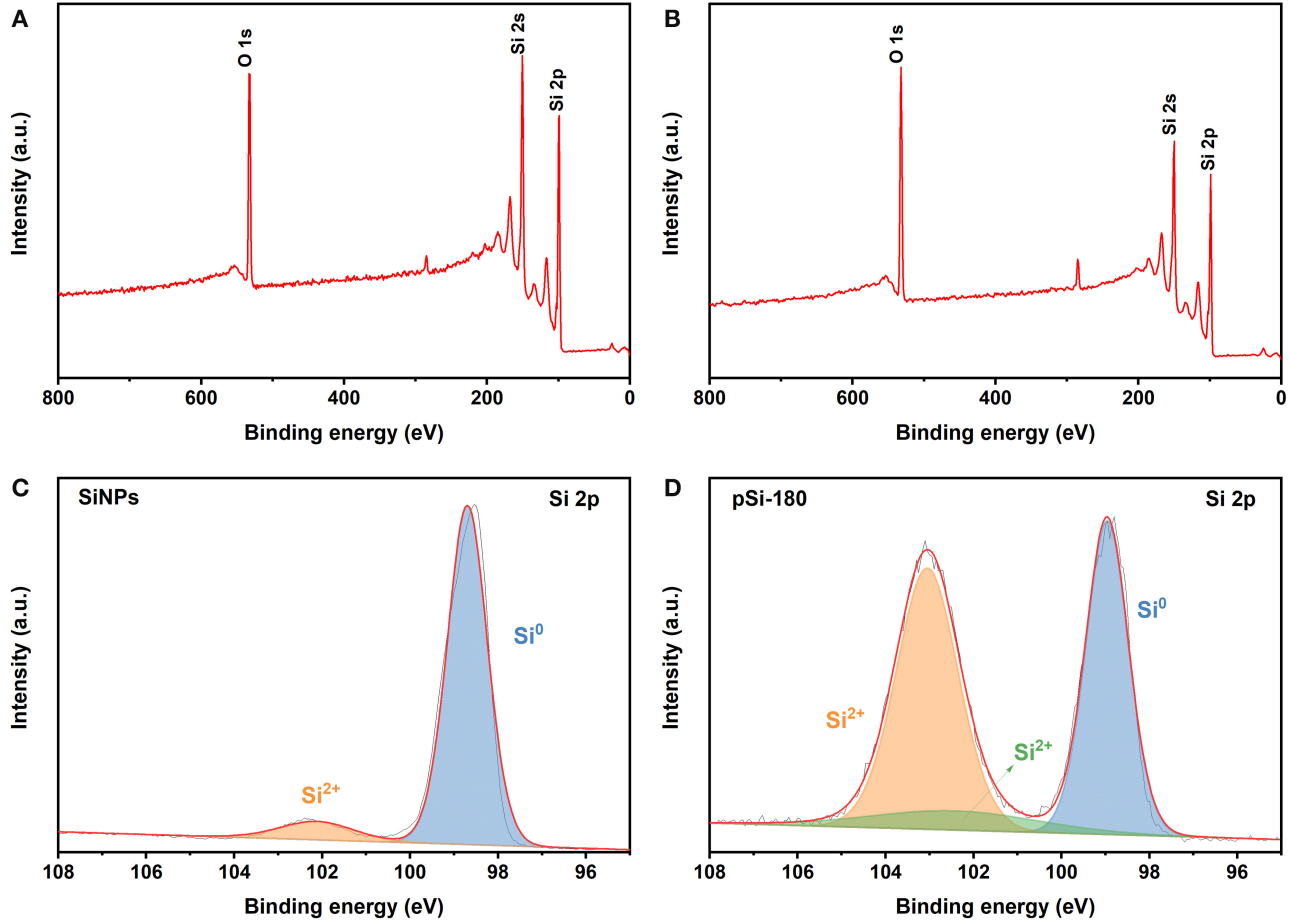
XPS spectra of SiNPs **(A)**, and pSi-180 **(B)**; High resolution XPS spectra of Si 2p for SiNPs **(C)**, and pSi-180 **(D)**.

**Figure 6:**
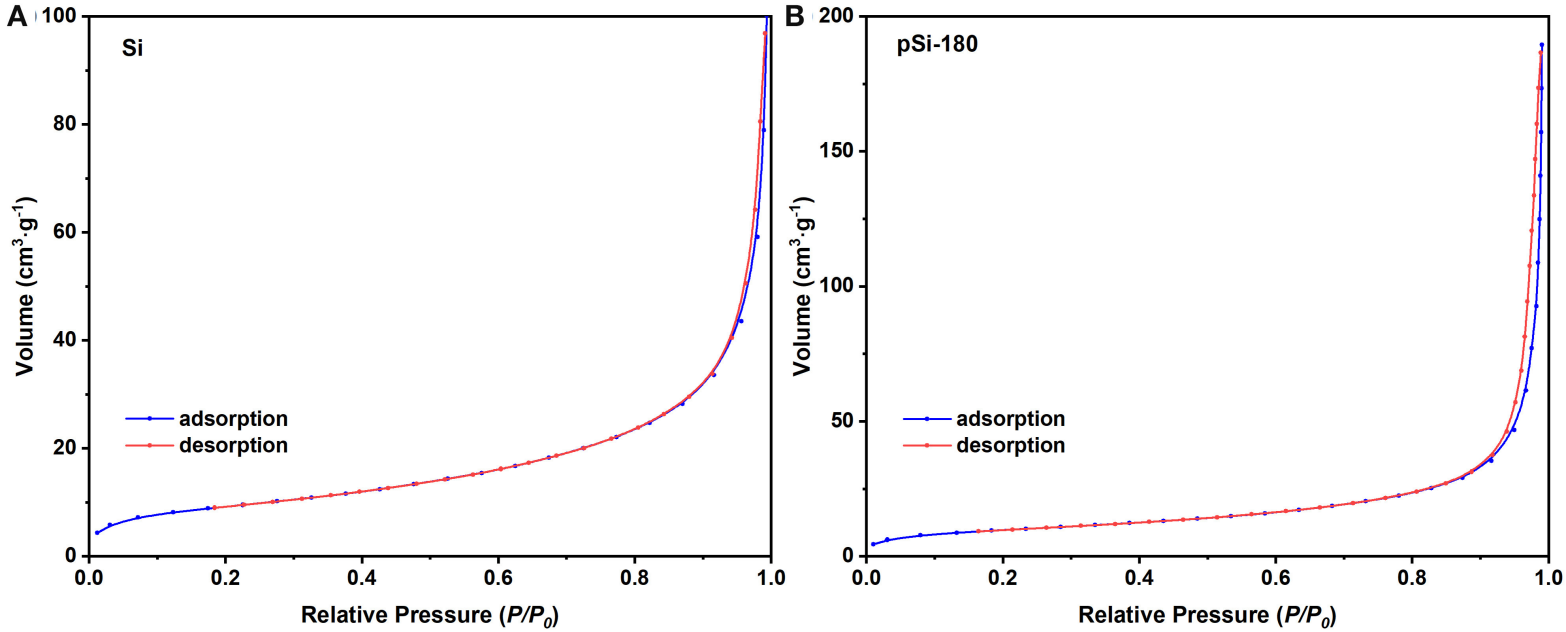
Nitrogen adsorption-desorption isotherms of **(A)** SiNPs, and **(B)** pSi-180.

**Figure 7:**
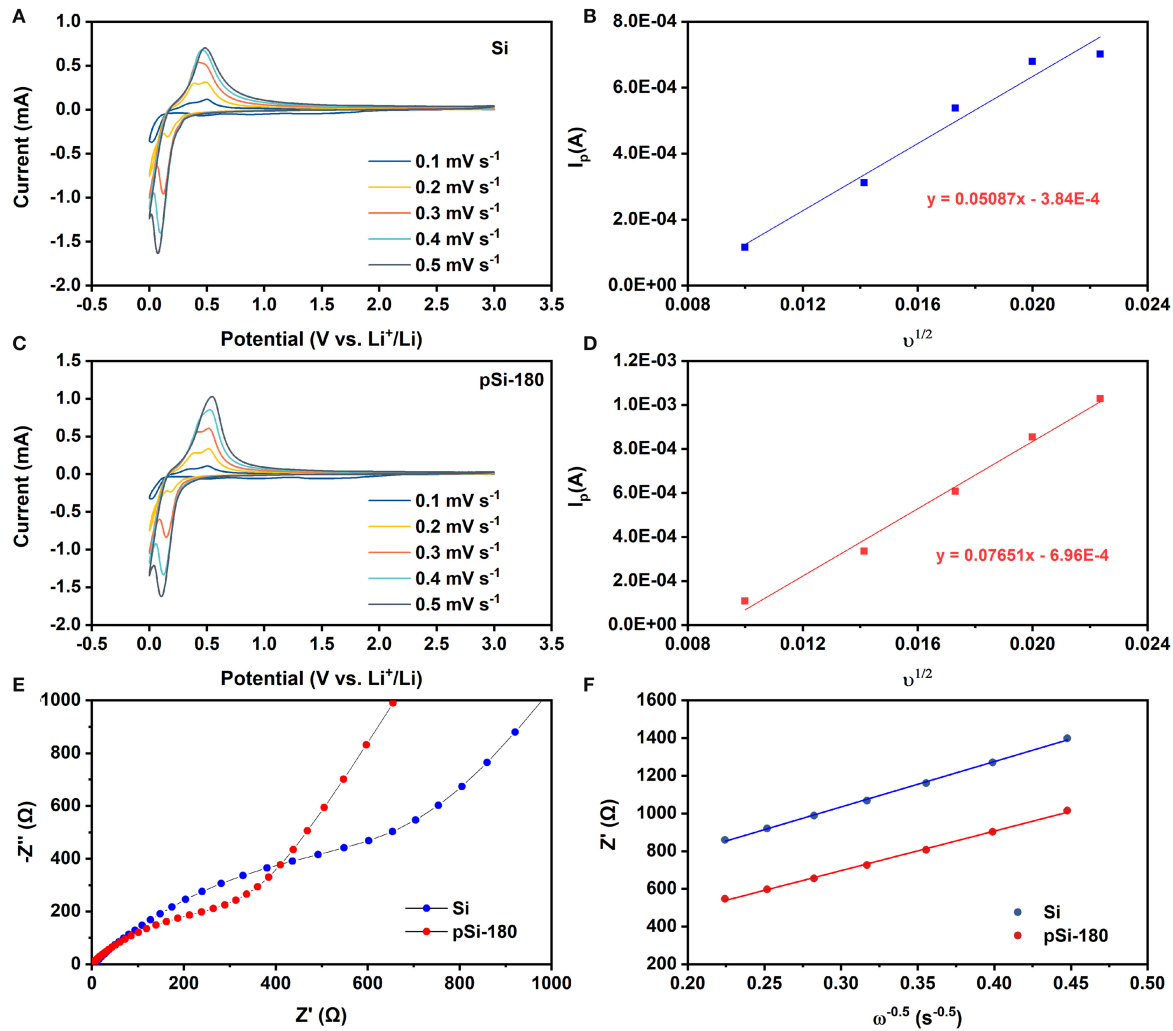
CV curves at different potential scan rates for SiNPs **(A)**, and pSi-180 **(C)**. Dependence of *I*_*p*_ on υ^1/2^ for SiNPs **(B)** and pSi-180 **(D)**; **(E)** EIS spectra of SiNPs and pSi-180 before cycling (fully delithiation); **(F)** The relationship between *Z*′ and ω^−0.5^ at low frequency.

As shown in Figure 2a, commercial SiNPs (20–120 nm) disorderly aggregate. After the spray drying process, SiNPs rearranged into the pomegranate-like structured second particles (Figures 2b,c, 1–7 μm, the particle size distribution maps are shown in Supplementary Figure 1). The EDS elemental mapping images in Figures 2d,e indicate that Si and O elements are uniformly distributed in the pSi-180 composite. The O element comes from the SiO_x_ coating layer, formed when SiNPs were in contact with hot air. During the lithiation process, SiO_x_ reacted with lithium-ions and generated Li_2_O and a series of lithium silicates, which could partly relieve the volume expansion (Wang et al., 2014).

In TEM images, the unique pomegranate-like structure is also observed (Figure 3a) and pores (bright area) are displayed in the composite, which could accommodate the volume expansion of a Si anode and contribute to the permeation of lithium-ion. Figures 3b–e, Supplementary Figure 2 shows the morphology of SiNPs and the pomegranate-like composites formed under different temperatures. The SiO_x_ layer can be observed on the surface of SiNPs, with thicknesses of 2, 5, 10, and ~15 nm for SiNPs, pSi-150, pSi-180, and pSi-210, respectively. With increasing temperature, the SiO_x_ coating layer becomes thicker. Figure 3f displays the selected area electron diffraction (SEAD) of the pSi-180. The four diffraction rings correspond to (111), (220), (311), and (400) crystal planes of Si, indicating that SiO_x_ was formed on the surface and SiNPs remains in the crystalline state inside.

The crystal phase states of the SiNPs, pSi-150, pSi-180, and pSi-210 were further analyzed by XRD (Figure 4A). The peaks at 28.4, 47.3, 56.1, 69.1, and 76.4° correspond to (111), (220), (311), (400), and (331) crystal planes of Si (PDF#77-2107), which is consistent with the SEAD result in Figure 3f. After the spray drying process, the peak positions of pSi-150, pSi-180, and pSi-210 are the same as those of SiNPs, indicating that the main crystal structure of SiNPs did not change. However, due to the formation of SiO_x_ on the surface, the peak intensity decreased with increasing drying temperature. According to the FT-IR spectra in Figure 4B, symmetric, and asymmetric Si-O-Si stretching vibrations are observed at 1,101 and 878 cm^−1^, respectively. As expected, the intensity of the two peaks of pSi-180 is higher than that of SiNPs due to the increasing amount of SiO_x_. The Raman spectra of SiNPs and pSi-180 are shown in Supplementary Figure 3. The peak at 514 cm^−1^, which belongs to Si-Si, has a decreased intensity after drying at 180°C due to the formation of SiO_x_.

XPS was used to further investigate the chemical composition and state of SiNPs and pSi-180. The peaks at 532, 150, and 99 eV are associated with O 1s, Si 2s, and Si 2p in Figures 5A,B. After the spray drying process, the strength of the O 1s peak increased. Figures 5C,D show the high-resolution XPS spectra of Si 2p for SiNPs and pSi-180, respectively. The peaks at 98.7 and 102.1 eV correspond to Si^0^ and Si^2+^, respectively. After the spray drying process, a new peak appears at the higher binding energy (103.0 eV), which is consistent with Si^4+^. The amount of SiO_x_ on the surface increased from 9.1 to 59.4% (calculated by CasaXPS), which could improve the cycling performance of the Si@SiO_x_ anode (Zhang X. L. et al., 2019).

Figure 6 shows the N_2_ adsorption-desorption isotherms of SiNPs and pSi-180. According to the Brunauer, Emmett, and Teller (BET) model, the specific surface area of SiNPs and pSi-180 was 33.5 and 35.0 m^2^ g^−1^, respectively. The improvement of the specific surface area was mainly due to the SiO_x_ on the surface of SiNPs, which could accommodate the agglomeration among SiNPs (Zheng et al., 2018). Based on the Barret, Joyner, and Halenda (BJH) model, the pore volume also increased after the formation of the pomegranate-like structure (0.15 to 0.29 cc g^−1^). During the spray drying process, SiNPs rearranged to the pomegranate-like structure, which could effectively alleviate the volume expansion stress during the repeated lithiation/delithiation process. Furthermore, the unique structure is conducive to the penetration of electrolyte and the diffusion of lithium-ion (Yu et al., 2018).

To investigate the lithium-ion storage in the pomegranate-like structured Si@SiO_x_ anode, CV was performed at different rates. As shown in Figures 7A,C, the peak at ~0.17 V in the reduction process are ascribed to the alloying process between Li^+^ and Si (Si Li_22_Si_5_). The anodic peaks at 0.33 V and 0.53 V are related to the reversible process (Li_22_Si_5_ amorphous Si). Although the CV curves deliver a similar shape, the anodic peak intensity of pSi-180 is higher than that of SiNPs. According to the Sevcik equation (Equation 3), the lithium-ion diffusion coefficient ($D_{Li^{+}}$) for SiNPs (Figure 7B) and pSi-180 (Figure 7D) is 6.0^*^10^−10^ and 1.4^*^10^−9^ cm^2^ s^−1^, respectively, indicating that the pomegranate-like structured anode is conducive to the diffusion of lithium-ion (Feng et al., 2019; Luo et al., 2019; Mu et al., 2019).

(3) $$\begin{array}{r} I_{p}=2.69*10^{5}n^{3/2}AD_{0}^{1/2}\upsilon^{1/2}C_{0} \end{array}$$

where n is the number of reaction electrons, A is the specific surface area of the electrode, C is the concentration of lithium-ion.

To better understand the role of SiO_x_ and the pomegranate-like structure, EIS measurements were conducted between 10 mHz and 100 kHz. As shown in Figure 7E, Nyquist plots of SiNPs and pSi-180 consist of two parts before cycling: a semicircle in the high-frequency region corresponding to the charge transfer resistance (*R*_*ct*_) and an oblique line in the low frequency region associated with the lithium-ion diffusion impedance in the electrode (*W*_*o*_). The point of intersection between the semicircle and the X-axis in the high region represents the contact resistance (*R*_*s*_). According to the equivalent circuit shown in Supplementary Figure 4, the calculated *R*_*ct*_, and *R*_*s*_ for SiNPs decreased after spray drying at 180°C (*R*_*ct*_: 818.7 Ω → 235.2 Ω; *R*_*s*_: 6.0 Ω → 0.8 Ω). According to Equation 5, the slope in Figure 7F represents the Warburg impedance coefficient (σ_*w*_), whose square is inversely proportional to $D_{Li^{+}}$. Hence, the $D_{Li^{+}}$ of pSi-180 is slightly higher than that of SiNPs (calculated thorough EIS), which is in accordance with the result calculated through CV curves with different scan rates. The pomegranate-like structure could enhance the contact area between the active materials and electrolyte, thereby improving the charge transfer resistance and lithium-ion diffusion rate (Li et al., 2018; Luo et al., 2019; Wang B. et al., 2020).

(4) $$\begin{array}{l} D=0.5{\left(RT\right)}^{2}/{\left(n^{2}F^{2}A\sigma_{\text{w}}C\right)}^{2} \end{array}$$

(5) $$\begin{array}{l} Z^{\prime }=R_{s}+R_{ct}+R_{SEI}+\sigma_{w}\omega^{-0.5} \end{array}$$

where *R* is the gas constant, *T* is the ambient temperature, n is the number of reaction electrons, *F* is the Faraday constant, *A* is the specific surface area of the electrode, *C* is the concentration of lithium-ion, and σ_*w*_ is the Warburg impedance coefficient.

**Figure 8:**
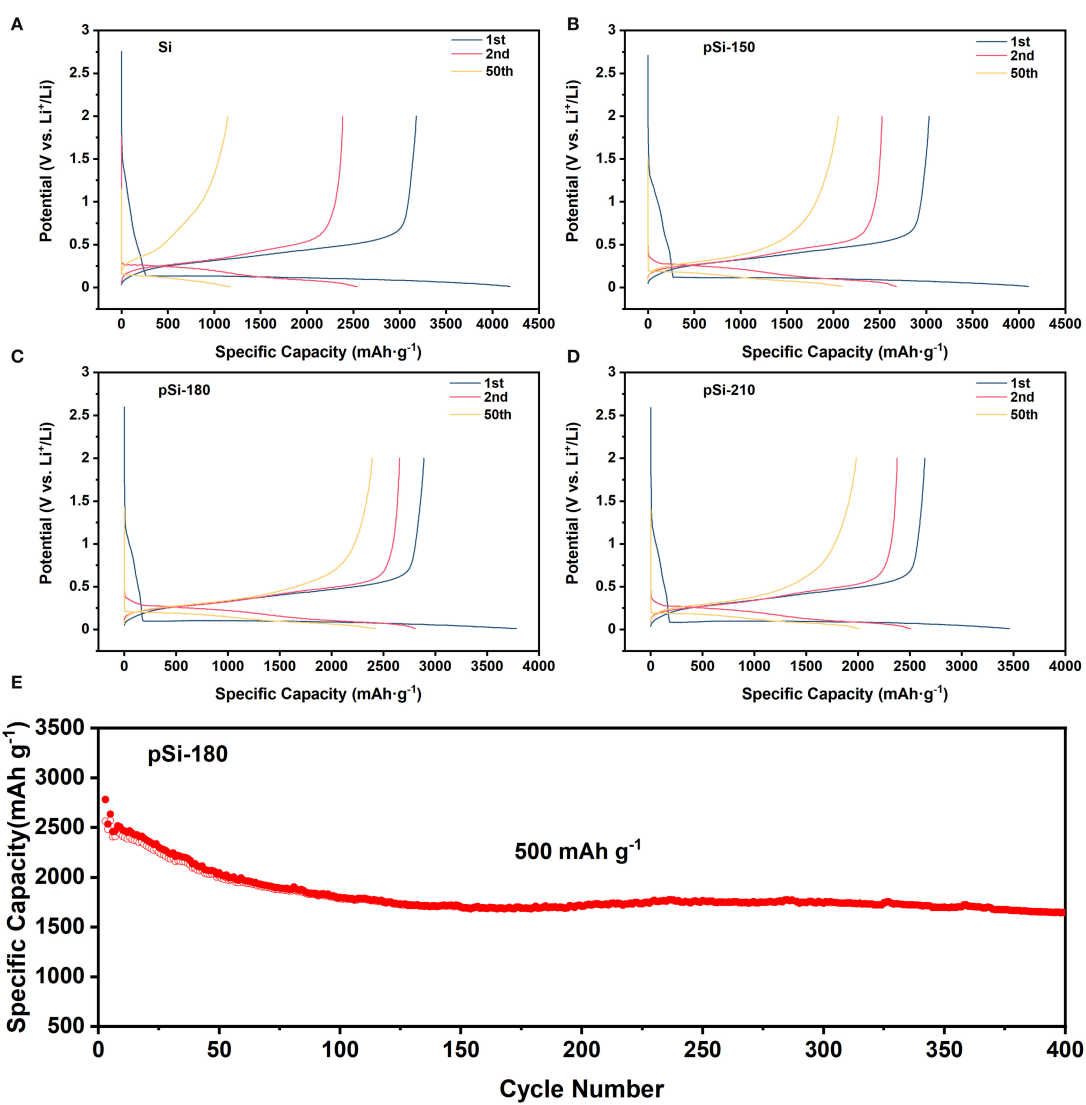
**(A–D)** Voltage-capacity curves for Si, pSi-150, pSi-180, and pSi-210 (100 mA g^−1^ for the first cycle and 200 mA g^−1^ for the following cycle); and **(E)** Long cycling performance of pSi-180 at 500 mA g^−1^.

Figures 8A–D shows the voltage-capacity curves of SiNPs, pSi-150, pSi-180, and pSi-210 at 100 mA g^−1^ for the first cycle, and 200 mA g^−1^ for the following cycles. In the first cycle, the charge/discharge specific capacity was 3179.0/4188.7, 3031.2/4103.6, 2891.2/3781.6, and 2644.6/3459.5 mAh g^−1^, for the four samples, respectively. And the Coulombic efficiency was 75.9, 73.8, 76.5, and 76.4%, respectively. With the increase in the drying temperature, the initial reversible specific capacity decreased for the surface of SiNPs, which was slightly oxidized to SiO_x_. In addition, Supplementary Figure 5 compares the voltage-capacity curves of the above samples in the first cycle, and it is observed that they exhibit similar charge/discharge plateaus. However, with increasing spray drying temperature, the voltage difference between charge, and discharge plateaus become larger due to the passivation of SiO_x_ on the surface (Zheng et al., 2018). In the second cycle, the reversible specific capacity was 2385.4, 2525.7, 2656.9, and 2378.5 mAh g^−1^ for SiNPs, pSi-150, pSi-180, and pSi-210, respectively. After 50 cycles, the reversible specific capacity was 1148.9, 2054.5, 2394.9, and 1984.7 mAh g^−1^, respectively. The 50th capacity retention of the above samples was 48.2, 81.3, 90.1, and 83.4%, respectively. With the aid of the SiO_x_ buffer layer and the unique pomegranate-like structure, the cycling stability was significantly improved.

Figure 8E shows the long cycling performance of pSi-180 at 500 mA g^−1^. After 100 cycles, the pSi-180 electrode delivers a high reversible specific capacity of 1786.3 mAh g^−1^. In the following cycles, pSi-180 remained stable and after 400 cycles it still presented a reversible specific capacity of 1642.9 mAh g^−1^ and remarkable cycling stability (0.09% decay per cycle). The rate capability was also tested at various current densities between 100 mA g^−1^ and 2,000 mA g^−1^, shown in Supplementary Figure 6. Reversible specific capacities of 2853.9, 2322.9, 2001.1, and 1519.8 mAh g^−1^ were obtained at 100, 500, 1,000, and 2,000 mA g^−1^, respectively. When the current density returned to 100 mA g^−1^ from the high rate, pSi-180 delivered 2686.7 mAh g^−1^, corresponding to a capacity recovery of 94.1%.

**Figure 9:**
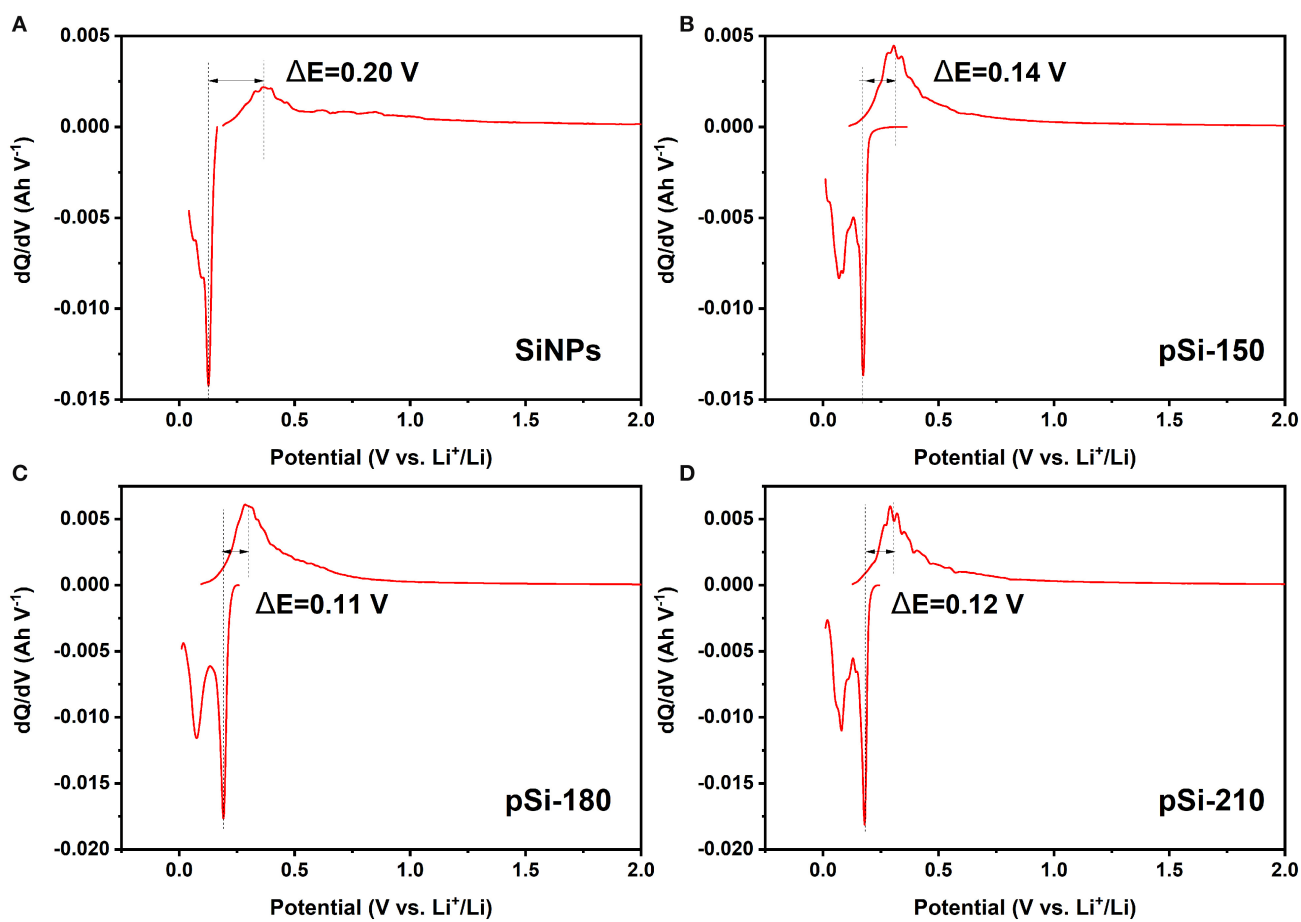
Differential capacity (dQ/dV) plots for Si **(A)**, pSi-150 **(B)**, pSi-180 **(C)**, and pSi-210 **(D)** after 50 cycles at 200 mA g^−1^.

As shown in Figure 9, the dQ/dV curves display a similar shape after cycling, which is consistent with the CV results. After introducing SiO_x_ and the formation of the pomegranate-like structure, the peak intensity of the structured composites increased compared to that of SiNPs, indicating better electrode activity. Additionally, the polarization potential difference between anodic and cathodic peaks for SiNPs, pSi-150, pSi-180, and pSi-210 after 50 cycles was 0.20, 0.14, 0.11, and 0.12 V, respectively. After the spray drying process, pomegranate-like structured composites suffered less polarization and delivered better lithium-ion lithiation/delithiation kinetics (Zhang Y. et al., 2019).

Similar works about pomegranate-like structured Si/C and Si@SiO_x_ anode are compared in Table 1. The pomegranate-like Si@SiO_x_ prepared in this work delivered a higher capacity than those of most Si-based anodes in earlier reports. In addition, spray drying technology is simple and convenient, which favors large-scale production. Moreover, water was used as a solvent, leading to an economical and environmentally friendly result.

**Table 1:** Cycling performance comparison of pomegranate-like structured Si/C and Si@SiO_x_ anode for lithium-ion batteries in similar reports.

**Anode material**	**Reversible specific capacity**	**Current density**	**References**
Pomegranate-like Si@C	1,204 mAh g^−1^ (100th)	0.5 A g^−1^	Mu et al., 2019
Pomegranate-like structure Si/C	581 mAh g^−1^ (200th)	0.2 A g^−1^	Shen et al., 2017
Pomegranate-like Si/N-G	1,100 mAh g^−1^ (150th)	0.1 A g^−1^	Lin et al., 2016
Pomegranate-like SiOx/C	1,024 mAh g^−1^ (200th)	0.5 A g^−1^	Yu et al., 2018
Core-bishell Si@SiO_x_@TiO_2−δ_	650 mAh g^−1^ (200th)	0.2 A g^−1^	Hu et al., 2019
Si–SiO_x_–C	1,035 mAh g^−1^ (200th)	0.1 A g^−1^	Lee et al., 2017
Hollow B-Si/SiO_x_@VN/PC	1,237 mAh g^−1^ (300th)	0.5 A g^−1^	Zhang X. L. et al., 2019
Pomegranate-like Si@SiO_x_	1,747 mAh g^−1^ (300th)	0.5 A g^−1^	This work

**Figure 10:**
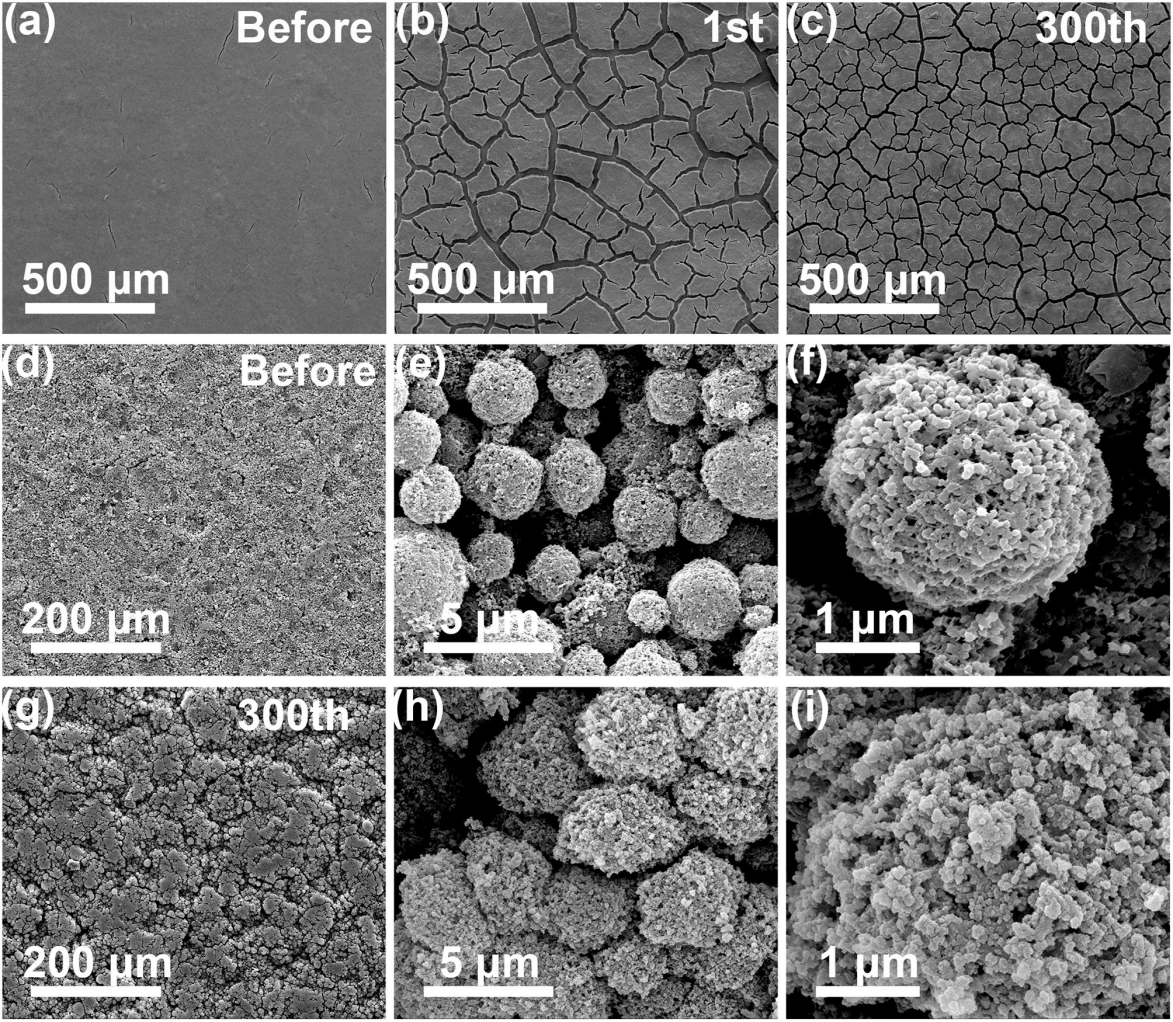
SEM images of Si electrode **(a)** before cycling; **(b)** after 1 cycle; **(c)** after 300 cycles; SEM images of pSi-180 electrode **(d–f)** before cycling; **(g–i)** after 300 cycles.

To investigate the structural stability during cycling, the morphology of the SiNPs and pSi-180 anode before and after cycling were observed by *ex-situ* SEM. As shown in Figures 10a–c, the surface of the SiNPs electrode before cycling was smooth with few cracks. After the first cycle, it was drastically fractured and cracked into smaller pieces after 300 cycles. Figures 10d–f shows the morphology of the pSi-180 electrode before cycling. The surface of the pSi-180 electrode was intact and the second particles maintained the pomegranate-like structure after coating on the current collector. After 300 cycles, the pSi-180 electrode (Figures 10g–i) suffered few cracks and the pomegranate-like structure maintained integrity except for a slight expansion. The cross profiles of SiNPs and pSi-180 anode before and after cycling were also obtained, as shown in Supplementary Figiure 7. After 300 cycles, the volume expansion ratio of SiNPs and pSi-180 was 243.2 and 21.6%, respectively. The structural stability difference between SiNPs and the pSi-180 electrode is mainly due to the SiO_x_ shell on the surface and its unique structure, which could accommodate the volume expansion during the repeated lithiation/delithiation process.

The characterization described and the analysis results of this study indicate that SiNPs could rearrange to a pomegranate-like structure during the spray drying process, in which SiNPs would react with oxygen in hot air and generate core-shell Si@SiO_x_ nanoparticles. The thickness of SiO_x_ could be tuned by adjusting the spray drying temperature. The SiO_x_ buffer material and the unique pomegranate-like structure could alleviate the volume expansion stress during the repeated lithiation/delithiation process. Thus, the pomegranate-like structured Si@SiO_x_ anode delivers a high reversible capacity after long cycling.

## Conclusion

The pomegranate-like structured Si@SiO_x_ was successfully prepared through a one-step spray drying process. By controlling the inlet air temperature, the thickness and oxidation degree of SiNPs could be tuned. In the series of the pomegranate-like Si@SiO_x_ composites, pSi-180 delivered a high discharge capacity of 1,747 mAh g^−1^ and outstanding long-cycling performance (0.13% loss per cycle) after 300 cycles at 500 mAg^−1^. In addition, pSi-180 presented a reversible capacity of 1519.8 mAh g^−1^ at 2 A g^−1^. This excellent electrochemical property comes from the SiO_x_ coating layer and the unique pomegranate-like structure, which can accommodate volume expansion during the repeated charge/discharge process. In this work, a one-step spray drying technology was used, which is conducive to the large-scale production of a Si anode. This method could be used in other nanoparticles to prepare pomegranate-like structured materials.

## Data Availability Statement

All datasets generated for this study are included in the article/Supplementary Material.

## Author Contributions

JL is responsible for conceptualization, methodology, and wrote the main manuscript text. WL, YQ, ZX, and MQ contributed immensely to the writing and discussion of the final manuscript. GP and YR are responsible for project administration and funding acquisition. In addition, they all approved the final manuscript for publication. All authors contributed to the article and approved the submitted version.

## Conflict of Interest

The authors declare that the research was conducted in the absence of any commercial or financial relationships that could be construed as a potential conflict of interest.
