# Supplementary material for: Pomegranate-Like Structured Si@SiOx Composites With High-Capacity for Lithium-Ion Batteries
Source: Front Chem. 2020 Sep 11;8:666. doi: 10.3389/fchem.2020.00666 (PMC7516033; doi:10.3389/fchem.2020.00666)
Supplement: Supplementary file 1 [file Table_1.DOCX]

**Pomegranate-like structured Si@SiO_x_ composites with high-capacity for lithium-ion batteries**

Jianbin Li^a, b^, Wenjing Liu^a^, Yingjun Qiao^a,b^, Gongchang Peng^a,*^, Yurong Ren^c,**^, Zhengwei Xie ^a***^, and Meizhen Qu^a^

^a^ Chengdu Institute of Organic Chemistry, Chinese Academy of Sciences, Chengdu 610041, PR China

^b^ University of Chinese Academy of Sciences, Beijing 100039, PR China

^c^ School of Materials Science and Engineering, Jiangsu Collaborative Innovation Center of Photovolatic Science and Engineering, Changzhou University, Changzhou,

213164, PR China





Fig. S1 The particle size distribution of SiNPs (a) and pSi-180 (b).


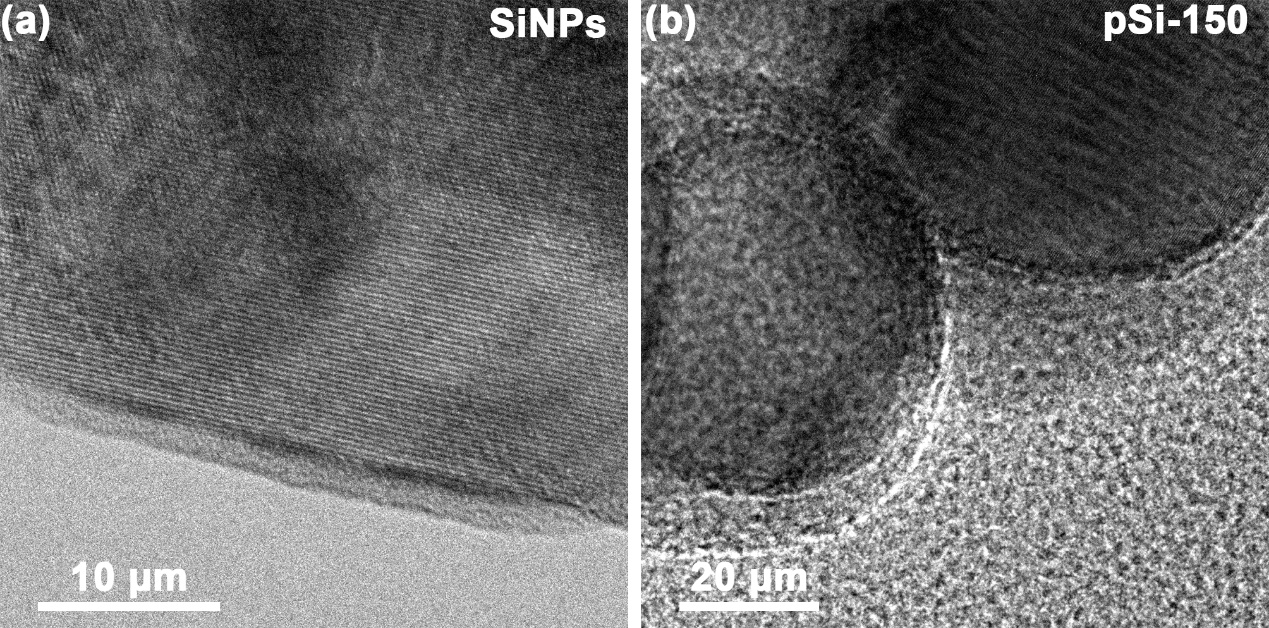


Fig. S2 The magnification TEM images of SiNPs (a) and pSi-150 (b).





Fig. S3 Raman spectra of SiNPs and pSi-180.


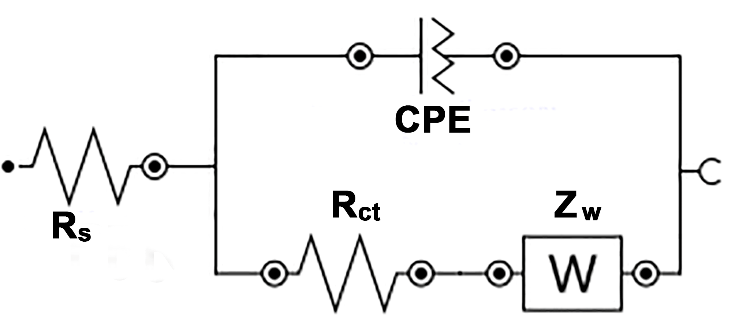


Fig. S4 Equivalent circuit before cycling.





Fig. S5 The voltage curves of SiNPs, pSi-150, pSi-180, and pSi-210 in the first cycle.





Fig. S6 Voltage profiles of pSi-180 electrode cycled at various rates from 0.1 to 2 A g^-1^ in a potential window from 0.01 to 2 V versus Li^+^/Li.


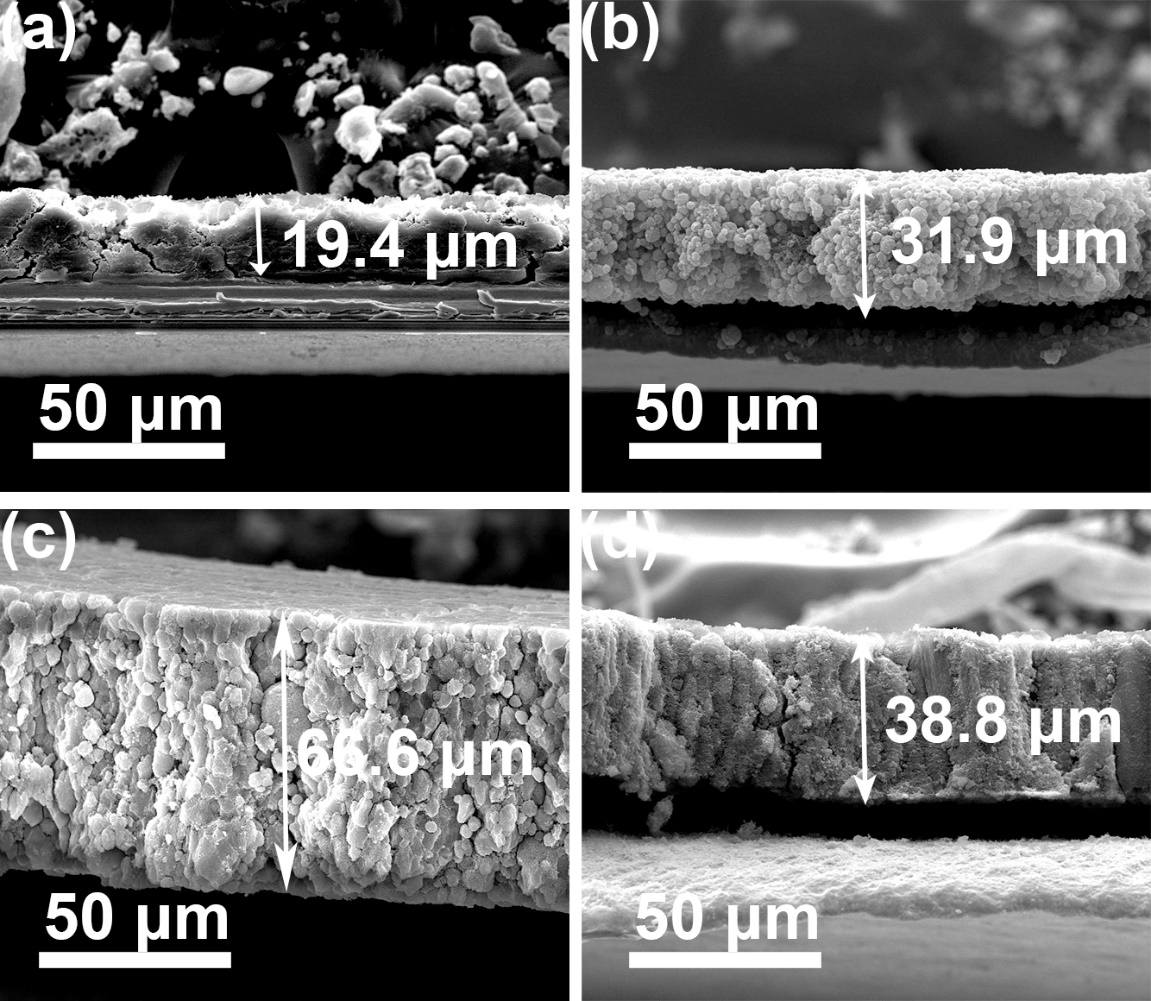


Fig. S7 The cross profiles of SiNPs and pSi-180 anode before and after cycling.
